# Verification labels for rovibronic quantum-state energy uncertainties

**DOI:** 10.1038/s41598-023-46665-0

**Published:** 2024-01-08

**Authors:** Péter Árendás, Tibor Furtenbacher, Attila G. Császár

**Affiliations:** 1 Budapest Business University, Budapest, Hungary; 2 Institute of Chemistry, ELTE Eötvös Loránd University, Budapest, Hungary; 3 HUN-REN–ELTE Complex Chemical Systems Research Group, Budapest, Hungary

**Keywords:** Computational chemistry, Physical chemistry, Applied mathematics

## Abstract

Transition wavenumbers contained in line-by-line rovibronic databases can be compromised by errors of various nature. When left undetected, these errors may result in incorrect quantum-state energies, potentially compromising a large number of derived spectroscopic data. Spectroscopic networks treat the complete set of line-by-line spectroscopic data as a large graph, and through a least-squares refinement the measured line positions are converted into empirical quantum-state energies. Spectroscopic networks also offer a highly useful framework to develop mathematical tools helping to identify possible errors and conflicts within the dataset. For example, wavenumber errors can be detected by checking for violations of the law of energy conservation. This paper describes a new graph-theory tool, which results in so-called verification labels for the quantum states. Verification labels help to express the vulnerability of a calculated empirical energy value and its uncertainty against possible wavenumber errors, providing complementary information to simple statistical uncertainties.

## Introduction

It is usual to represent the cumulative results of high-resolution spectroscopic studies of rovibronic transitions in the form of line lists^1,2^. Each assigned line of the list contains descriptive (quantum-number) information about the two quantum states the transition connects, along with additional physical quantities, such as wavenumber (position of the center of the line), Einstein-*A* coefficients (transition intensity), lineshapes, temperature-dependent self and foreign pressure broadenings and shifts, etc. Moreover, the physical quantities, most notably the wavenumber values, should also have accompanying uncertainty values in the line lists, as required both for measured and computed data^3^.

In the modern scientific era information about rovibronic lines has two principal sources: they come either from measurements or from first-principles computations. Some may say that rovibronic information can also come from effective Hamiltonian (EH) determinations; while this is true, EH parameters are considered here to be representations of measured data and not as sources of spectroscopic data. In this paper, we do not distinguish transitions based on their origin, everything discussed here applies to both experiment and theory. As a significant restriction, in what follows only wavenumber values and their uncertainties are discussed.

The most notable derived information from measured transitions are the energies of the quantum states. Using the transition wavenumbers and their uncertainties in a line list, one can calculate quantum-state energy values and a corresponding uncertainty. Assuming that the experimental information is correct and accurate, energies provide a compact representation of the measurements, complementing the traditional EH approach.

Unfortunately, it often happens that for some lines in the line list, the wavenumber value, its uncertainty, or both are incorrect. This can occur due to measurement errors, human mistakes, or various other reasons. These errors may be captured by appropriate mathematical tools during the analysis of part of or preferably the complete line list, but some errors might avoid detection. These incorrect values influence the calculated energy values and their uncertainties much beyond the local environment.

As a result, it is important to augment the uncertainties derived for the quantum state energies with additional information, providing extra, complementary verification of the numerical values. In this paper, verification labels are introduced to supplement the energy and uncertainty value pairs. The verification label of a quantum-state energy expresses the vulnerability of its uncertainty against possible wavenumber errors within the given line list.

The structure of this paper is as follows. The section “Theoretical background” presents the theoretical background, centered around spectroscopic networks (SN)^4^, a graph representation of a set, preferably a complete set, of spectroscopic data. This connection between spectroscopy and graph theory has already made possible the creation of various mathematical tools aiding the detection, and subsequent correction, of data issues in high-resolution molecular spectroscopy^5–11^. The section “Verification of line lists” elaborates on the relationship between the consistency and the correctness of line-by-line spectroscopic databases. The section “Labeling scheme” introduces a new labeling scheme. The verification label of quantum state *X* is based primarily on the verification metric, *V*(*X*), and secondarily on a graph property of *X*, both defined within this section. The section “A practical example: the W2020 database of water transitions” demonstrates verification labeling on the example of the W2020 line-by-line spectroscopic database^12^ of the H$_2^{~16}$O molecule. The section “Conclusions” contains the conclusions of the paper.

## Theoretical background

For convenience, Table 1 highlights the most important symbols and terms used in this section and the rest of the paper.

**Table 1:** Symbols and terms used in “Theoretical background” section.

$w_i$	‘True’ transition wavenumber value (unknown)
${\hat{w}}_i$	A wavenumber value in a line list, approximating $w_i$
$u_i$	The uncertainty value of ${\hat{w}}_i$ in the line list
${\hat{W}}_i$	The wavenumber interval ${\hat{w}}_i \pm u_i$
$w'_i$	A wavenumber value based on all ${\hat{W}}_i$ intervals of the line list. They are selected to yield zero-sum cycles
$W'$	The set of the $w'_i$ values of a line list
$u'_i$	Uncertainty value, based on $u_i$, subject to increase to achieve consistency of the line list
*E*(*X*)	Energy value of quantum state *X*
*U*(*X*)	Uncertainty of quantum state *X*
$P_X$	The set of the edges of the shortest path from the root to *X* using the $u'_i$ edge weights

### Correctness of wavenumber entries

Let us index the transitions of a line list *L* with *i*, and denote the ‘true’ transition wavenumber, that is the line center position, of the *i*th transition by $w_i$. The unknown $w_i$ is estimated by the wavenumber value in the line list, denoted by ${\hat{w}}_i$. The wavenumber ${\hat{w}}_i$ is reported together with a measurement uncertainty $u_i$. Let ${\hat{W}}_i=({\hat{w}}_i-u_i, {\hat{w}}_i+u_i)$ be the *wavenumber interval* of the *i*th transition. The wavenumber interval should include the $w_i$ value with a probability of at least $95\%$, in other words, $P(w_i \in {\hat{W}}_i) \ge 0.95$. This is in accordance with the convention to report uncertainties with a $2\sigma$ uncertainty, where $\sigma$ denotes the standard deviation. Let us call a wavenumber interval ${\hat{W}}_i$ for which $w_i \notin {\hat{W}}_i$ an *incorrect wavenumber interval*.

Since the $w_i$ values are not known, it is not straightforward to ascertain whether ${\hat{W}}_i$ is correct or not. However, one could take external information related to ${\hat{W}}_i$ into consideration and come up with a decision whether to consider ${\hat{W}}_i$ correct or incorrect.

A trivial example for an incorrect wavenumber interval ${\hat{W}}_i$ is when ${\hat{w}}_i+u_i < 0$, as wavenumber values must be positive reals. Spectroscopic information systems, for example, those based on the MARVEL (Measured Active Rotational Vibrational Energy Levels) technique^13–15^, use several advanced supporting methods to assess the correctness of the wavenumber intervals of a line list.

Some of these methods are based on the graph representation of high-resolution rovibronic spectroscopic data, called a spectroscopic network^16^. The new labeling introduced in this paper also relies on this representation. Thus, let us continue by covering the required theory about spectroscopic networks.

### Spectroscopic networks

Spectroscopic networks offer a highly useful representation of line-by-line spectroscopic data, especially when they come from a large number of sources of different origin and of different accuracy. The *spectroscopic network* of a molecule is a graph *G*(*V*, *E*), in which the vertex set *V* represents the rovibronic quantum states of the molecule, and the edge set *E* corresponds to allowed transitions between the quantum states. Certain physical quantities can be utilized as weight functions; most notably, quantum state energies as vertex weights, and transition intensities and wavenumbers as edge weights. The term ‘spectroscopic network’ is not an exact definition of a graph: it has to be specified, based on the given application, which weights to use, or, for example, whether it is defined to be a directed or an undirected graph.

**Figure 1:**
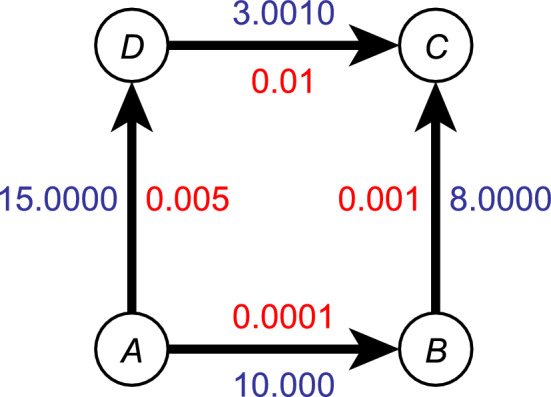
Example of a small spectroscopic network (SN), with four quantum states, *A*, *B*, *C*, and *D*, and four transitions between the states. The blue (outside) numbers represent transition wavenumbers, while the red (inside) numbers represent the corresponding transition uncertainties. As usual in rovibrational spectroscopy, the unit is cm$^{-1}$.

Figure 1 depicts a small SN which has only four quantum states and four transitions among the states. The blue numbers, outside of the graph, represent transition wavenumbers, while the red numbers, inside of the graph, are the respective transition uncertainties, both in units of cm$^{-1}$. We will continue referring to Fig. 1 as more definitions and SN properties are introduced.

The size of a SN depends on the underlying spectroscopic data set. While one can construct a network with only a few quantum states and a few transitions among them, like in Fig. 1, usual applications of SNs are characterized by inputs of large size. For example, the H$_2{}^{16}$O line list in the HITRAN spectroscopic information system^1^ has 319,887 lines (transitions) that span 14,130 quantum states. Therefore, one of the main challenges of designing graph algorithms for application in spectroscopy comes from the sheer number of vertices and edges. For example, in practice, it is not recommended to use the adjacency matrix representation of SNs. Adjacency matrixes have size of $|V| \times |V|$, where the large |*V*| values involved in the typical calculations make even storing the matrix challenging, if not impossible. It is advised to use the adjacency list representation instead, that is storing the neighbours of each vertex in a list, yielding a much smaller-sized data structure.

Spectroscopic networks can be defined either as directed or undirected graphs. In the directed case, edges are directed from the lower-energy quantum state of the transition towards the higher-energy quantum state (the transition occurs in absorption). In Fig. 1, for example, the directed edge $e_{AB}$ from *A* to *B* corresponds to a transition from the lower-energy quantum state *A* to the higher-energy quantum state *B*. The quantum state of the molecule that is defined to have the zero energy value is the *root* of the spectroscopic network.

A line list may contain multiple lines of the same transition; for example, if multiple measurements are available. These are represented by parallel edges in the SN.

For a graph *G*(*V*, *E*), a *path* $P\subseteq E$ of length $k-1$ is an edge set $\{e_1, e_2,..., e_{k-1}\} \subseteq E$ for which there exists a vertex set $\{v_1,..., v_k\} \subseteq V$ such that for $1 \le i \le k$ the endpoints of $e_i$ are $v_i$ and $v_{i+1}$. In this paper, the direction of the edges in a path is defined to be irrelevant; in Fig. 1, there are two paths from *A* to *D*: one is $\{e_{AD}\}$ and the other is $\{e_{AB}, e_{BC}, e_{DC}\}$. If edge weights are considered, then a shortest path between two vertices is the edge set with the smallest sum of their weights. For example, the shortest path in Fig. 1 between vertices *A* and *C*, using the uncertainties as weights, is $A\rightarrow B\rightarrow C$, with a weight sum of $0.001\,1$.

A *cycle* $C \subseteq E$ is a path if $v_1 = v_k$ and $k>2$. If the edge $e_i$ does not participate in any cycles in the graph, then it is called a *bridge*^9^.

A graph is 2-*edge-connected* if there exists at least two edge-disjoint paths between any two of its vertices (i.e., at least two paths such that there is no edge that appears in both paths). For a graph *G*(*V*, *E*) with a root vertex, let us denote the maximal 2-edge-connected subgraph that contains the root by $G'(V',E')$. Note that $V' \subseteq V$, $E' \subseteq E$, the edge set $E'$ does not contain any bridges, and any edge in $E'$ participates in at least one cycle in $G'$.

Almost without exception, SNs based on experimental data are bipartite graphs, a result of the standard rovibronic selection rules governing transitions among the quantum states^5^. According to this, the number of edges of any cycle of the SN must be even. More explicitly, the smallest cycle in a spectroscopic network formed by dipole-allowed one-photon transitions has four edges (explaining the choice for Fig. 1).

### Law of energy conservation

An important property of a directed spectroscopic network is that the sum of the $w_i$ wavenumbers along each cycle of the graph, with the weights that are travelled backwards counted as negative, is equal to zero. This property of the cycles of SNs is guaranteed by the quantum nature of the transitions, embodied in the *law of energy conservation*^8,11^.

Recall that in a line list we do not have the unknown $w_i$ values, only the ${\hat{W}}_i$ wavenumber intervals, and the wavenumber interval is incorrect when $w_i \notin {\hat{W}}_i$. Thus, the use of the law of energy conservation in this environment is as follows: one should be able to select a wavenumber from each ${\hat{W}}_i$ interval such that using these wavenumbers, the aforementioned sum along all cycles is zero. If such a wavenumber selection is not possible, then the line list contains at least one incorrect wavenumber. The reverse, however, is not true: even if such a wavenumber selection exists, there could be still incorrect wavenumbers present in the line list.

The law of energy conservation plays a pivotal role in investigations revealing incorrect wavenumber intervals in a line list. It allows to compare wavenumber intervals of multiple lines to each other, providing an excellent external information source for deciding about the correctness of a wavenumber interval.

To formalize this concept, let a *wavenumber selection function*, $f(L) = W'$, where *L* is a line list, define a set of wavenumbers $W'=\{w'_1, w'_2,...\}$ for all edges of the spectroscopic network such that for all cycles in the graph, using the $w'_i$ wavenumbers as edge weights, and with the weights that are travelled backwards counted as negative, is equal to zero. Additionally, for any wavenumber selection function, $W'$, it is required that $w'_i = w'_j$ if $e_i$ and $e_j$ are parallel edges (i.e., their endpoints are the same). Note that the variable of the function *f* is not the set of all ${\hat{W}}_i$ wavenumber intervals in the line list but the line list itself; this definition allows the use of any kind of information contained in the line list in the selection of the $w'_i$ values.

If there exists a wavenumber selection $W'$ such that $\forall w'_i \in W': w'_i \in {\hat{W}}_i$, that is, if all selected wavenumbers lie in their corresponding wavenumber intervals, both the wavenumber selection and the line list are called *consistent*. Otherwise, if for a line list no consistent wavenumber selection exists, the line list is called *inconsistent*.

There is a consistent underlying line list behind Fig. 1, Table 2 proves this by showing a particular wavenumber selection. Note that if a line list is consistent, then practically there is an infinite number of consistent wavenumber selections. Therefore, additional preferences must be taken into consideration when selecting wavenumber values. Within the usual, time-proven MARVEL protocol^14,15^, for example, the $w'_i$ wavenumbers are determined by minimizing $\sum _i |\frac{{w'_{i}}-{\hat{w}}_{i}}{u_{i}}|^2$.

**Table 2:** A wavenumber selection, proving the consistency of the underlying line list of the spectroscopic network of Fig. 1.

edge	$\hat{w_i}$	$u_i~~$	$w'_i$	$|\hat{w_i}-w'_i|$
$A$ $\rightarrow$ $B$	$10.000\,0$	$0.000\,1$	$10.000\,0$	0
$B$ $\rightarrow$ $C$	8.000	0.001	8.000	0
$A$ $\rightarrow$ $D$	$15.000\,0$	0.005	$15.000\,0$	0
$D$ $\rightarrow$ $C$	$~3.001\,0$	0.01	$3.000\,0$	0.001

Note that in each row $u_i \ge |\hat{w_i}-w'_i|$. Similar to Fig. 1, the unit is cm$^{-1}$.

The verification labeling method introduced in this paper requires a consistent line list as its input. Therefore, although the method is demonstrated on a MARVEL-based spectroscopic data set, we disregard *how* the data was processed by MARVEL, and how the exact MARVEL energies were calculated.

### Calculation of the energy values

Hereafter, let us consider the set $\{(w'_1, u'_1), (w'_2, u'_2),...\}$. This set does not contain any parallel edges, and the $u'_i$ uncertainties are either the original $u_i$ uncertainties, or some of them might have been increased to make the line list consistent.

If we have a wavenumber selection $W'$, then the sum of the wavenumbers $w'_i$ on the edges $e_i$ of a path from the root to another vertex *X*, with wavenumbers of edges travelled in the reverse direction counted as negative, gives the estimation for the energy value *E*(*X*). Note that *E*(*X*) is a function of the quantum state *X*; not to be confused with *E*, that is without any variables, which denotes an edge set. The estimated energy value does not depend on the path: any path from the root to *X* gives the same energy value for *E*(*X*).

Similar to the uncertainties of the wavenumbers, the calculated quantum-state energies also need to be augmented with well-defined uncertainties. Let $P_X \subseteq E$ be the shortest path from the root to quantum state *X* using the $u'_i$ uncertainties as edge weights. Let us define the uncertainty of the energy value of *X* as

1 $$\begin{aligned} U(X)=\sum _{\forall i: e_i \in P_X} u'_i. \end{aligned}$$

**Table 3:** Energy values and uncertainties for Fig. 1, based on the wavenumber selection of Table 2 and assuming the role of the root quantum state for vertex *A*.

*X*	Shortest uncertainty path	*E*(*X*)	*U*(*X*)
*A* (root)	–	0	0
*B*	$A \rightarrow B$	$10.000\,0$	$0.000\,1$
*C*	$A \rightarrow B \rightarrow C$	$18.000\,0$	$0.001\,1$
*D*	$A \rightarrow D$	$15.000\,0$	0.005

Similar to Fig. 1, the unit is cm$^{-1}$.

Utilizing the wavenumber selection of Tables 2 and 3 shows the energy values and the corresponding uncertainties for the SN of Fig. 1, where the root quantum state is vertex *A*. Note that the energy value estimation depends on the $w'_i$ values, but the uncertainties are independent of them.

## Verification of line lists

### Consistency does not imply correctness

If a line list is consistent, one might, albeit mistakenly, assume that it implies that all wavenumber intervals in the line list are correct. This is not true. Let us demonstrate in a simple example that consistency does not imply correctness.

The line list corresponding to Fig. 1 has already been shown to be consistent. There, the wavenumber of the $A \rightarrow B$ transition is ${\hat{w}}_{AB} = 10.000\,0$, with an uncertainty of $u_{AB} = 0.000\,1$. If we assume that they form a correct wavenumber interval, we have $w_{AB} \in (10 \pm 0.000\,1)$.

Let us denote the line list of Fig. 1 by $L_{\text{orig}}$. Let $L_{\text{mod}}$ be the line list we obtain from $L_{\text{orig}}$ after changing a wavenumber value: let ${\hat{w}}_{AB} = 10.01$. Observe that we have increased the original ${\hat{w}}_{AB}$ value by a number that is much larger than the corresponding uncertainty: $0.01 > u_{AB}$. However, and this is the source of a lot of problems, the modified line list is still consistent, as proven by the wavenumber selection in Table 4. We obtain a contradiction if we assume that the wavenumber intervals of the consistent $L_{\textrm{mod}}$ line list are also correct: $w_{AB} \in (10 \pm 0.000\,1)$ and $w_{AB} \in (10.01 \pm 0.000\,1)$ cannot be true simultaneously.

**Table 4:** Wavenumbers ${\hat{w}}_i$ and uncertainties $u_i$ of the line list $L_{\textrm{mod}}$, and a wavenumber selection (the ${\hat{w}}'_i$ values) that proves the consistency of $L_{\textrm{mod}}$.

edge	$\hat{w_i}$	$u_i$	$w'_i$	$|\hat{w_i}-w'_i|$
$A$ $\rightarrow$ $B$	$10.010\,0$	$0.000\,1$	$10.010\,0$	0
$B$ $\rightarrow$ $C$	8.000	0.001	8.000	0
$A$ $\rightarrow$ $D$	$15.000\,0$	0.005	$15.000\,0$	0
$D$ $\rightarrow$ $C$	$3.001\,0$	0.01	$3.010\,0$	0.009

**Table 5:** Energy values and the corresponding uncertainties based on the line list $L_{\textrm{mod}}$.

*X*	$E_{\textrm{orig}}(X)$	$E_{\textrm{mod}}(X)$	difference	*U*(*X*)
*A* (root)	0	0	0	0
*B*	$10.000\,0$	$10.010\,0$	0.01	$0.000\,1$
*C*	$18.000\,0$	$18.010\,0$	0.01	$0.001\,1$
*D*	$15.000\,0$	15.000	0	0.005

The ‘difference’ column contains the $|E_{\textrm orig}(X)-E_{\textrm{mod}}(X)|$ values.

Moreover, as illustrated in Table 5, this error propagates to the energy values. Most notably, observe that the uncertainty of *B* is $U(B)=0.000\,1$, but the difference between the energy value of *B* in the two cases is two orders of magnitude larger.

This artificial increase of ${\hat{w}}_{AB}$ is similar to the typical wavenumber error that originates in measurement errors or human typing mistakes. Therefore, it is necessary to develop a mathematical tool that helps to detect, assess, and handle this phenomenon in line-by-line spectroscopic datasets.

### Wavenumber error detection

Can we increase or decrease wavenumber values of a consistent line list arbitrarily without losing consistency? Fortunately, for transitions that take part in at least one cycle, the law of energy conservation does provide a bound. To illustrate this, note that we cannot increase ${\hat{w}}_{AB}$, for example, by 100 (i.e., one hundred cm$^{-1}$): ${\hat{w}}_{AB}=110.000$ would make it impossible to select wavenumber values from the four wavenumber intervals to obtain the zero sum along the cycle.

Therefore, as the law of energy conservation interconnects the transitions of the line list based on cycles, one can use this to predict the maximum artificial increase for each line that does not violate this law. Unfortunately, this cannot be applied for the bridges of the spectroscopic network: here, ${\hat{w}}_i$ can be increased by any positive real number without breaking consistency.

### The $d_i$ threshold of transitions

First, let us discuss wavenumber errors. For this, let us define the *threshold* of the *i*th transition $e_i$, denoted by $d_i$, to be the greatest number for which the line list $\{(w'_1, u'_1), ... , (w'_i\pm (u'_i+d_i), u'_i), ... (w'_m, u'_m)\}$ is consistent. Since consistency is based on cycles, let us restrict this definition to the non-bridge edges of the SN.

The $d_i$ values can be calculated deterministically with arbitrary accuracy, though with an enormous calculation runtime, by running the wavenumber selection over and over, varying the $d_i$ candidate values. As this route is unfeasible, let us define a ${\hat{d}}_i$ upper bound for each $d_i$ as follows:

2 $$\begin{aligned} {\hat{d}}_i = \max _{x}\{(0, u'_1), ... , (u'_i+ x, u'_i), ... (0, u'_m)\}, \end{aligned}$$

for which $\{(0, u'_1),..., (u'_i+ x, u'_i),... (0, u'_m)\}$ is consistent.

Observe that ${\hat{d}}_i \ge d_i$ by definition. For the the *i*th transition in the line list, its ${\hat{d}}_i$ value expresses that an arbitrary increase or decrease that is larger than ${\hat{d}}_i$ will be detected when checking the consistency of the line list.

Note that an arbitrary increase or decrease that is much smaller than ${\hat{d}}_i$ might also be detected when cheking consistency. Capturing these errors depends both on the line list itself and the wavenumber selection function used. Here, the mathematical statement is that errors that are larger than ${\hat{d}}_i$ will be detected at all times.

This ${\hat{d}}_i$ value can be calculated efficiently. Let us denote the shortest path between the endpoints of $e_i$ in the graph $G(V, E\setminus \{e_i\})$, using $u'_i$ edge weights, by $S(e_i)$. This can be done, for example, using Dijkstra’s algorithm^17^ (the name Dijkstra is the reason for the notation of $d_i$). Note that, by definition, it is an edge set: $S(e_i) \subseteq E$. Then, $\hat{d_i}$ is the length of $S(e_i)$:

3 $$\begin{aligned} \hat{d_i} = \sum _{\forall j: e_j \in S(e_i)} u'_j. \end{aligned}$$

For example, let us consider the estimation of $d_{AB}$ in Fig. 1 (note that Table 2 already proves the consistency of the underlying line list). Here, we have ${\hat{d}}_{AB} = 0.015\,1$, because $S(e_{AB}) = \{e_{AD}, e_{DC}, e_{BC}\}$.

Note that (a) the estimation of $d_i$ depends on the line list; therefore, it is a property inherited from the underlying transitions of the SN, and (b) the ${\hat{d}}_i$ values can already be used as standalone pieces of information, describing the line list’s own capability in detecting incorrect wavenumbers. Moreover, one can also calculate ${\hat{d}}_{XY}$ before adding the very first *X*–*Y* transition to the line list, offering a priori information.

Now, one can ask what happens when we calculate the uncertainty of an energy value according to the linear formula, Eq. (1)? We take a sum of $u'_i$ uncertainties, but each non-bridge edge $e_i$ has already a ${\hat{d}}_i$ value. The next subsection transfers the concept of the ${\hat{d}}_i$ values to the quantum states.

### The *V*(*X*) verification of quantum states

We would like to extend the idea presented in the section “The d_i_ threshold of transitions” from transition wavenumbers to quantum-state energies. Briefly, if the *U*(*X*) uncertainty of quantum state *X* is calculated by taking the sum of some $u_i$ uncertainties (see the section “Calculation of the energy values”), then let us use the corresponding ${\hat{d}}_i$ values to express the vulnerability of *U*(*X*) against incorrect wavenumbers in a new *V*(*X*) value.

We only have ${\hat{d}}_i$ values for non-bridge edges; thus, let us restrict the calculation of *V*(*X*) to the maximal 2-edge-connected subgraph of the spectroscopic network which contains the root quantum state. Recall that the vertex set of this component is denoted by $V'$.

Let us define the *verification* *V*(*X*) of quantum state $X \in V'$ as follows:

4 $$\begin{aligned} V(X)=\sum _{\forall i:\, e_i \in P_X } {\hat{d}}_i + \sum _{\begin{subarray}{c} \forall i: \,e_i \in P_X, \\ \not \exists j \ne i : \,\, e_i \in S(e_j) \end{subarray}} u'_i. \end{aligned}$$

Briefly, *V*(*X*) is equal to *U*(*X*) plus the uncertainties along each $S(e_i)$ path $\forall i:\,e_i \in P_X$. Observe that we count each $u'_i$ either zero or one time: if $\exists \,e_i \in P_X: e_i \in S(e_j), \, i \ne j$, then $e_i$ appears only once in the sum that defines *V*(*X*). The rightmost column of Table 3 shows the verifications of the energy values of Fig. 1.

### Small-uncertainty 4-edge-cycle density along $P_X$

We can add a second layer when describing the vulnerability of *E*(*X*) by further inspecting its uncertainty-defining shortest path $P_X$. Intuitively, if each $e_i \in P_X$ participates in a large number of 4-edge-cycles (the shortest possible cycle in a bipartite SN) that all have small combined uncertainties, then *X* is less vulnerable to errors, than with only a few cycles, or with cycles formed by edges with large uncertainties.

To capture this phenomenon, let $c(e_i)$ denote the number of 4-edge-cycles in which $e_i$ participates, where the sum of the other three uncertainties is smaller than $10 \cdot {\hat{d}}_i$. Then, let $k(X) = \min _{e_i \in P_X} c(e_i)$.

An efficient method to find 4-edge-cycles in a large graph is shown in Ref.^18^. The *k*(*X*) value now holds information about the density of small-uncertainty 4-edge-cycles along $P_X$; thus, it can also be used in the labeling.

## Labeling scheme

Based on the section “Verification of line lists”, we can construct a labeling scheme of the quantum states, expressing the vulnerability of their energy value and its uncertainty against wavenumber errors occurring in the line list. First, considering the typical range of uncertainty values, given in cm$^{-1}$, let us introduce labels based on the *V*(*X*) verification values according to Table 6. Based on the section “Small-uncertainty 4-edge-cycle density along P_X_”, the second step is to pick a reasonable empirical threshold for *k*(*X*), then assign a ‘+’ symbol after the A–F label of the quantum state *X* if *k*(*X*) is greater than this threshold.

**Table 6:** Labels and the corresponding *V*(*X*) magnitudes.

Label of *X*	*V*(*X*) magnitude (cm$^{-1}$)
A	$10^{-7}$ or smaller
B	$10^{-6}$
C	$10^{-5}$
D	$10^{-4}$
E	$10^{-3}$
F	$10^{-2}$ or greater

The MARVEL spectroscopic information system uses Gaussian uncertainty propagation^19^ when calculating energy value uncertainties. This formula for the uncertainty of *X*, where the subscript refers to the squaring of the uncertainties, is

5 $$\begin{aligned} U_2(X)=\sqrt{\sum _{\forall i: e_i \in P_X} (u'_i)^2}, \end{aligned}$$

with the $P_X$ path that minimizes this value. Note that this path may be different from the $P_X$ of the linear *U*(*X*) formula. However, because of the monotonicity of the square root function, it is enough to find the $P_X$ path for $U_2(X)$ to run Dijkstra’s algorithm using not $u'_i$ but $(u'_i)^2$ edge weights.

To make the *V*(*X*) verification comparable to these values, let us define

6 $$\begin{aligned} {\hat{d}}_{i,2} = \sum _{\forall j: e_j \in S(e_i)} (u'_j)^2, \end{aligned}$$

and

7 $$\begin{aligned} V_2(X)=\sqrt{\sum _{\forall i:\, e_i \in P_X } {\hat{d}}_{i,2} }+ \sum _{\begin{subarray}{c} \forall i: \,e_i \in P_X, \\ \not \exists j \ne i : \,\, e_i \in S(e_j) \end{subarray}} (u'_i)^2, \end{aligned}$$

with the $P_X$ path that minimizes $U_2(X)$.

We use the same labeling when using $V_2(X)$ as the one that has been introduced for *V*(*X*) in Table 6.

## A practical example: the W2020 database of water transitions

Hereby we discuss the verification labels corresponding to an empirical line-by-line database of water transitions, called W2020^12^, developed by two of the present co-authors. In order to get our data in line with the usual conventions of molecular spectroscopy, we opted to use the uncertainty and verification formulas that use Gaussian uncertainty propagation, see Eqs. (5) and (6). We decided to assign a ‘+’ symbol after the A-F labels of quantum state *X* (see Table 6), if $k(X) \ge 3$.

**Table 7:** Distribution of verification labels corresponding to the H$_2^{~16}$O entries of the W2020 line list.

Label	Number of quantum states (incl. with ‘+’)	$k(X) \ge 3$
A	32	0
B	46	0
C	552	5
D	2725	22
E	8663	128
F	4805	68
N/A	2459	−

The ‘N/A’ row represents the quantum states with no label assigned: these quantum states are not in the maximal 2-edge-connected component that contains the root; thus, they are not subject to this labeling (see the section “The *V(X)* verification of quantum states”). Additionally, the root also received a N/A label. Note that a quantum state *X* received a ‘+’ in its label if $k(X) \ge 3$.

Table 7 shows the distribution of the verification labels corresponding to the H$_2^{~16}$O entries of the W2020 line list^12^. Out of the 19,282 quantum states that define the line list, 57 are not reachable from the root (they are in what we call ‘floating components’), and an additional 2401 nodes can only be reached through at least one bridge. Thus, $V_2(X)$ and $U_2(X)$ values were calculated for $19,282-57-2401-1=16,823$ quantum states (the root is also omitted).

Note that the uncertainties of the 2401 quantum states which can be reached only through at least one bridge can still be calculated using transition wavenumbers of the line list, i.e., *U*(*X*) and $U_2(X)$ both require just the presence of one path. It is just their verification *V*(*X*) and $V_2(X)$ that is not defined, due to the lack of the necessary presence of at least two edge disjoint paths leading to them from the root. To calculate the energy value of the 57 quantum states in the floating components external sources are required, most notably, EH or first-principles energy-level data.

The first observation related to Table 7 is that very few energy levels have the labels A or B. This is understandable, as there are only a relatively small number of very accurate measurements, with uncertainties on the order of a few kHz, in the W2020 database for H$_2^{~16}$O and the number of energy levels which participate in a cycle of high accuracy measurements is even smaller.

Second, the situation of the quantum states with a lower-quality verification label should be addressed. It must be emphasized that an ‘F’-labeled state can still have a correct wavenumber interval, it just cannot be verified more accurately using the other transitions in the line list.

The third important observation is that the most frequently occuring accuracy in the W2020 database is $\sim$ $10^{-3}$ cm$^{-1}$. This is the accuracy of results obtained with the technique of Fourier-transform infrared spectroscopy, used for the largest number of transition measurements.

The quantum states with the smallest and largest $V_2(X)$ values are shown in Tables 8 and 9, respectively. Note that the phenomenon that multiple $U_2$ and $V_2$ values are equal might happen quite easily; for example, in Fig. 1, quantum states *B*, *C*, and *D* all have the same *V* or $V_2$ value.

The results in Table 8 show the effectiveness of the Spectroscopic-Network-Assisted Precision Spectroscopy (SNAPS) procedure^19,20^, used for the design of measurements yielding line center positions with just a few kHz accuracy in the near infrared region (in fact around 7000 cm$^{-1}$).

Table 10 shows the quantum states with the smallest and largest $V_2(X)/U_2(X)$ ratios. The largest ratios show that not all of the transitions measured via the SNAPS procedure are part of cycles formed by transition with high (kHz) accuracy. While an effort was made to create cycles when the measurements reported in Ref.^19^ were designed, the high cost of these kHz-accuracy line center position measurements prevented to obtain an even larger number of cycles.

**Table 8:** Quantum states with the smallest $V_2(X)$ values in the W2020^12^ line list of H$_2^{~16}$O.

Quantum state	$U_2(X)$	$V_2(X)$
1 0 1 1 0 1	$1.03 \times 10^{-7}$	$2.24 \times 10^{-7}$
2 0 0 1 1 1	$1.40 \times 10^{-7}$	$2.24 \times 10^{-7}$
0 0 0 2 2 0	$1.44 \times 10^{-7}$	$2.24 \times 10^{-7}$
2 0 0 3 1 3	$1.75 \times 10^{-7}$	$6.30 \times 10^{-7}$
2 0 0 3 3 1	$1.83 \times 10^{-7}$	$6.30 \times 10^{-7}$
0 0 0 4 0 4	$2.02 \times 10^{-7}$	$6.30 \times 10^{-7}$
0 0 0 4 2 2	$2.19 \times 10^{-7}$	$6.30 \times 10^{-7}$
0 2 1 5 4 1	$2.46 \times 10^{-7}$	$6.30 \times 10^{-7}$
0 0 0 6 0 6	$2.69 \times 10^{-7}$	$6.30 \times 10^{-7}$
0 0 0 5 1 5	$3.99 \times 10^{-7}$	$6.30 \times 10^{-7}$
2 0 0 4 4 0	$4.16 \times 10^{-7}$	$6.30 \times 10^{-7}$
0 0 0 5 3 3	$4.29 \times 10^{-7}$	$6.30 \times 10^{-7}$

Three of the entries have the overall smallest value of $2.24 \times 10^{-7}$, then another 9 quantum states have the second smallest $V_2(X)$ value, $6.30 \times 10^{-7}$.

**Table 9:** Quantum states with the largest $V_2(X)$ values in the W2020^12^ line list.

Quantum state	$U_2(X)$	$V_2(X)$
11 1 3 1 1 1	$0.042\,438\,2$	$0.073\,511\,9$
11 1 3 2 1 1	$0.042\,438\,2$	$0.073\,511\,9$
14 2 1 1 1 1	$0.042\,438\,2$	$0.073\,511\,9$
14 2 1 2 1 1	$0.042\,438\,2$	$0.073\,511\,9$
15 1 1 1 1 1	$0.042\,438\,2$	$0.073\,511\,9$
15 1 1 2 1 1	$0.042\,438\,2$	$0.073\,511\,9$
15 2 0 1 0 1	$0.042\,438\,2$	$0.073\,511\,9$
16 0 1 1 1 1	$0.042\,438\,2$	$0.073\,511\,9$
16 0 1 2 1 1	$0.042\,438\,2$	$0.073\,511\,9$
16 1 0 1 0 1	$0.042\,438\,2$	$0.073\,511\,9$
16 1 0 2 2 1	$0.042\,438\,2$	$0.073\,511\,9$
17 0 0 1 0 1	$0.042\,438\,2$	$0.073\,511\,9$

A total of 12 quantum states have the largest $V_2(X)$ value, $0.073\,511\,9$.

**Table 10:** Quantum states with the largest and smallest $V_2(X)/U_2(X)$ values in the W2020^12^ line list.

Quantum state	$U_2(X)$	$V_2(X)$	$V_2(X)/U_2(X)$
1 0 1 25 1 25	$0.010\,191$	$0.011\,162\,9$	1.095
1 0 1 25 0 25	$~0.010\,24$	$0.011\,407\,1$	1.114
2 0 1 25 1 25	$0.020\,093\,3$	$0.022\,931\,9$	1.141
2 0 1 25 0 25	$0.020\,093\,3$	$0.023\,051\,5$	1.147
0 1 1 20 7 13	$0.010\,056\,5$	$0.011\,643\,4$	1.158
.	.	.	.
2 0 0 1 0 1	$2.70 \times 10^{-7}$	$0.000\,300\,17$	1111.577
0 1 3 5 1 5	$8.76 \times 10^{-7}$	$0.001\,000\,05$	1141.254
0 1 3 4 0 4	$8.50 \times 10^{-7}$	$0.001\,001\,31$	1177.979
0 1 3 3 1 3	$8.50 \times 10^{-7}$	$0.001\,063\,05$	1250.266
0 1 3 6 0 6	$8.87 \times 10^{-7}$	$0.001\,365\,96$	1540.047

**Figure 2:**
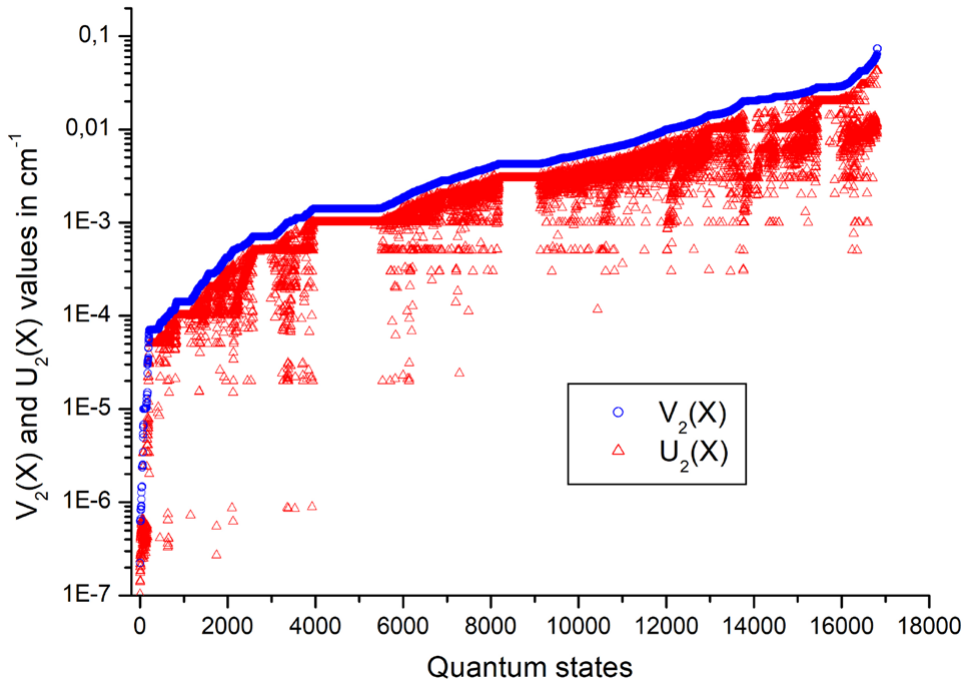
$U_2(X)$ and $V_2(X)$ values in the W2020 line list, sorted in ascending order of the $V_2(X)$ values.

Finally, Fig. 2 shows the $U_2(X)$ and $V_2(X)$ values, sorted in the ascending order of the $V_2(X)$ values. It can be seen immediately from this figure that the $V_2(X)$ values are always larger than the corresponding $U_2(X)$ values. Most of the time the $U_2(X)$ value is close to the $V_2(X)$ value, showing that the $U_2(X)$ value is a good approximation of the uncertainty of the empirical energy level. Nevertheless, there are several cases where $U_2(X)$ is much smaller than $V_2(X)$. This typically occurs when a part of a path of very accurate transitions is surrounded by large uncertainty transitions in the spectroscopic network. The path itself can still produce a small $U_2(X)$ value, but it cannot be verified as accurately, due to the lack of transitions of similarly good uncertainty surrounding the entirety of the path. Thus, these energies might still be accurate, but even a single transition with a wavenumber error may cause a large inaccuracy for them in this line list.

## Conclusions

It is found quite frequently that line-by-line spectroscopic databanks contain wavenumber errors, even after rigorous data cleansing and the usual careful analysis of the experimental line positions. These errors propagate directly into the empirical energies of the quantum states, leading most often to incorrect energy uncertainty intervals. The present paper advocates a method that helps to assess the effect wavenumber errors have on empirical energy values. The method developed relies on the spectroscopic network representation of the rovibronic line list.

Line lists should contain transition information that adheres to the law of energy conservation. The main idea of this paper is that each transition has a certain (unknown) threshold $d_i$ in the network, and any error larger than this threshold is detected after checking the violation of the law of energy conservation. An efficient estimation of ${\hat{d}}_i$ can be achieved by using Dijktra’s algorithm in the spectroscopic network.

By merging these thresholds an estimation can be made for the effect of undetected wavenumber errors in the line list. In this paper, it is called the *V*(*X*) verification of the quantum-state uncertainty. The *V*(*X*) value is to be used alongside the energy value *E*(*X*) and its uncertainty *U*(*X*) of quantum state *X*. If a quantum state *X* has a small uncertainty *U*(*X*) and also a small verification *V*(*X*), then one can trust much more the correctness of the energy value than in the case when *V*(*X*) is significantly higher than *U*(*X*). In the latter case, the uncertainty might still be accurate, but a single transition with a large wavenumber error can make it inaccurate.

Based on these verification values, a labeling scheme was introduced. This paper also shows the application of this labeling method to the W2020 dataset of the H$_2{}^{16}$O molecule.

## Data Availability

The datasets used and/or analysed during the current study available from the corresponding author on reasonable request. The codes used in this study can be found at https://respecth.elte.hu/verificationLabels.php.
